# Book Reviews

**Published:** 1911-02

**Authors:** 


### BOOK REVIEWS

A MANUAL OF NURSING. By Margaret Frances Donahoea, formerly Supt. of Nurses and Principal of Training School, Philadelphia General Hospital. Illustrated. 489 pages. Published by D. Appleton & Co., New York.

In this volume is briefly described the methods of caring for a patient, and the various duties of the trained nurse. The work is well written and contains much valuable information for nurses—to whom we heartily recommend it.

THE PRACTITIONER'S VISITING LIST for 1911. An invaluable pocket-sized book containing memoranda and data important for every physician, and ruled blanks for recording every detail of practice. The Weekly, Monthly and 30-Patient Perpetual contain 32 pages of data and 160 pages of classified blanks. The 60-Patient Perpetual consists of 256 pages of blanks alone. Each is one wallet-shaped book, bound in flexible leather, with flap and pocket, pencil with rubber, and calendar for two years. Price by mail, postpaid, to any address, $1.25. Thumb-letter index, 25 cents extra. Descriptive circular showing the several styles sent on request. Lea & Febiger, Publishers, Philadelphia and New York.

It is issued in four styles to meet the requirements of every practitioner: "Weekly," dated for 30 patients; "Monthly," undated for 120 patients per month; "Perpetual," undated, for 30 patients weekly per year, and "60 Patients," undated, for 60 patients weekly per year.

The text portion of The Practitioner's Visiting List for 1911 has been thoroughly revised and brought up to date. It contains among other valuable information a scheme of dentition; tables of weights and measures and comparative scales; instructions for examining the urine; diagnostic table of eruptive fevers; incompatimles, poisons and antidotes; directions for effecting artificial respiration; extensive table of doses; an alphabetical table of diseases and their remedies, and direction for ligation of arteries. The record portion contains ruled blanks of various kinds, adapted for noting all details of practice and professional business.

---

PRESCRIPTION WRITING AND FORMULARY. By John M. Swan, M. D., Associate Professor of Clinical Medicine, Medico-Chirurgical College of Philadelphia. 32mo of 185 pages. Philadelphia and London: W. B. Saunders Company, 1910. Flexible leather, $1.25, net.

Swan has written an excellent little book in which is condensed various useful combinations of well-known and well-tried drugs. Such works as this should greatly assist the practitioner in preparing elegant and useful prescriptions.

A TEXT-BOOK OF MODERN MATERIA MEDICA AND THERAPEUTICS. By A. A. Stevens, A. M., M. D., Professor of Therapeutics and Clinical Medicine, Woman's Medical College, Philadelphia. Octavo of 675 pages. Cloth, $3.50 net. W. B. Saunders & Co., Philadelphia.

This successful work by Stevens has now reached its 5th edition and represents the latest therapeutic advances. It may well be taken as a reliable guide in modern therapeutics. The text has undergone a thorough revision and the new remedies included in its pages.

---

DAWN OF THE FOURTH ERA IN SURGERY AND OTHER SHORT ARTICLES. By Robert T. Morris, M. D., Professor of Surgery, New York Post Graduate Medical School and Hospital. 12mo. of 145 pages. Philadelphia and London: W. B. Saunders Company, 1910. Artistically bound. $1.25 net.

This interesting little volume is full of human interest and useful observations. The "fourth era" in medicine is the method of treating disease by assisting nature and physiologic processes without meddlesome interference. Every surgeon should read the chapter entitled, "The Hand of Iron in the Glove of Rubber."

---

PHYSICAL EXAMINATION AND DIAGNOSTIC ANATOMY. By Charles B. Slade, M. D., Instructor in Physical Diagnosis, University and Bellevue Hospital Medical College, New York. 12mo. of 146 pages. Illustrated. Philadelphia and London: W. B. Saunders Company, 1910. Cloth. $1.25 net.

That diagnosis is the keystone upon which all treatment should be based is a truth too evident to permit of denial. This is the most difficult part of medicine, yet upon it must always depend the treatment. To paraphrase Mark Twain's expression, one might well say that the difference between right treatment and nearly right treatment is the "difference between lightning and the lightning bug." This work deals chiefly with the normal individual and does not take up specific diseases. It will afford an excellent basis upon which to build careful diagnosis and thus eliminate "lightning-bug treatment."

---

BIOLOGY: GENERAL AND MEDICAL. By Joseph McFarland, M. D., Professor of Pathology and Bacteriology, Medico Chirurgical College of Philadelphia. Octavo of 440 pages, with 160 illustrations. Philadelphia and London: W. B. Saunders Company, 1910. Cloth, $1.75 net.

The author has presented in this work the peculiar nature and interesting reactions of "living substance," and traces it from its probable beginnings through its many differential and complexities. The work is not ultra-technical so that the reader of average intelligence can comprehend most of its teachings without difficulty.

---

A MANUAL OF DISEASES OF THE NOSE, THROAT AND EAR. By E. Baldwin Gleason, M. D., Professor of Otology at the Medico-Chirurgical College, Philadelphia. Second revised edition. 12mo. of 563 pages, profusely illustrated. Philadelphia and London: W. B. Saunders Company, 1910. Flexible leather, $2.50 net.

This manual supplies the student and practitioner the essential facts of rhinology, laryngology, and otology in as concise form as possible. The entire subject of anatomy, physiology and pathology of the upper respiratory tract and ear have received careful consideration and thus renders the work complete for study or reference.

